# An exploration of the methods to determine the protein‐specific synthesis and breakdown rates in vivo in humans

**DOI:** 10.14814/phy2.14143

**Published:** 2019-09-08

**Authors:** Lars Holm, Kasper Dideriksen, Rie H. Nielsen, Simon Doessing, Rasmus L. Bechshoeft, Grith Højfeldt, Marcus Moberg, Eva Blomstrand, Søren Reitelseder, Gerrit van Hall

**Affiliations:** ^1^ Institute of Sports Medicine and Department of Orthopedic Surgery M Bispebjerg Hospital Copenhagen Denmark; ^2^ Department of Biomedical Sciences Faculty of Health and Medical Sciences University of Copenhagen Copenhagen Denmark; ^3^ School of Sport, Exercise and Rehabilitation Sciences University of Birmingham Birmingham United Kingdom; ^4^ Aastrand Laboratory Swedish School of Sport and Health Sciences Stockholm Sweden; ^5^ Department of Physiology and Pharmacology Karolinska Institutet Stockholm Sweden; ^6^ Clinical Metabolomics Core Facility Department of Clinical Biochemistry, Rigshospitalet Copenhagen Denmark

**Keywords:** Amino acid recycling, deuterated alanine, deuterated water, fractional breakdown rate, fractional synthesis rate, protein turnover, stable isotope

## Abstract

The present study explores the methods to determine human in vivo protein‐specific myofibrillar and collagenous connective tissue protein fractional synthesis and breakdown rates. We found that in human myofibrillar proteins, the protein‐bound tracer disappearance method to determine the protein fractional breakdown rate (FBR) (via ^2^H_2_O ingestion, endogenous labeling of ^2^H‐alanine that is incorporated into proteins, and FBR quantified by its disappearance from these proteins) has a comparable intrasubject reproducibility (range: 0.09–53.5%) as the established direct‐essential amino acid, here L‐ring‐^13^C_6_‐phenylalanine, incorporation method to determine the muscle protein fractional synthesis rate (FSR) (range: 2.8–56.2%). Further, the determination of the protein breakdown in a protein structure with complex post‐translational processing and maturation, exemplified by human tendon tissue, was not achieved in this experimentation, but more investigation is encouraged to reveal the possibility. Finally, we found that muscle protein FBR measured with an essential amino acid tracer prelabeling is inappropriate presumably because of significant and prolonged intracellular recycling, which also may become a significant limitation for determination of the myofibrillar FSR when repeated infusion trials are completed in the same participants.

## Introduction

The study of protein mass adaptation in whole body and limb, in structures such as skeletal muscle and tendons, and in various tissues and organs during health and disease requires knowledge of the dynamics of protein turnover (Garlick and Millward 1972; Millward et al. 1975; Young 1976; Matthews 1993). Thus, valid measures of protein turnover involving both protein synthesis and breakdown rates are mandatory. These measures are possible with the use of stable isotopically labeled amino acid tracers. Using such tracers, many approaches exist and care must be taken with designing complex studies involving repeated tracer exposure in, for example, cross‐over studies.

Two distinct tracer principles, the tracer dilution and the direct incorporation, form the basis for existing approaches to measure protein turnover rates. The tracer dilution principle measures amino acid tracer dilution in whole body (1‐pool) or across a tissue‐bed with sampling of arterial and tissue‐draining venous blood (2‐pools) or within pools closer to a tissue of interest by the addition of sampling sites: the interstitial compartment by microdialysis and the intracellular compartment by obtaining biopsies (Barrett et al. 1987; Biolo et al. 1992; Gore et al. 2007). The tracer dilution method is a direct measure of amino acid flux rates in and out of pools, hence allows a concomitant estimate of both protein synthesis and breakdown rates, and is frequently applied in muscle physiological research (Barrett et al. 1987; Gelfand and Barrett 1987; Biolo et al. 1994). However, the tracer dilution principle is protein unspecific and thus limited when the purpose is to investigate the changes in protein turnover of a specific protein or protein group. In this case, the alternative option, the direct incorporation method also known as the amino acid tracer precursor–product method (Garlick et al. 1973; Toffolo et al. 1993), allows a direct measure of a fraction of proteins or even single protein synthesis rates. This, however, can be limited by the requirement of sampling the proteins of interest, and it should be in sufficient quantities (e.g., Balagopal 1998; Jaleel et al. 2008). Basically, the approach is that protein synthesizing cells are exposed to known amounts of an amino acid tracer and by measuring the abundance of tracer being incorporated into the protein between two time points (Garlick et al. 1975; Golden and Waterlow 1977), a direct average protein synthesis rate (the fractional synthesis rate (FSR)) is determined as a gross mean between the two time points. A protein‐specific approach – comparable to the FSR method – to determine the protein degradation is also warranted (Chinkes 2005; Holm and Kjaer 2010). We previously presented a method where the rate of loss of protein‐bound tracer is followed as a reflection of the fractional breakdown rate (FBR) determined exactly on the same protein pools as used for assessing the FSR (Holm et al. 2013). The principle of this prelabeling approach is to first label proteins of interest by administering deuterated water (^2^H_2_O). Deuterium atoms will be transferred from water to several metabolites via various metabolic pathways (Neese et al. 2002; Strawford et al. 2004; Busch et al. 2006). Deuterium‐labeled amino acids are incorporated into proteins during *de novo* synthesis, thus labeling the proteins. Hereafter, a period of time has to pass to allow ^2^H_2_O as the label donor to disappear from the precursor pool to secure no further re‐incorporation of deuterium‐labeled amino acids into protein. Once the ^2^H_2_O enrichment has disappeared, the loss of labeled amino acids from the protein pool will be a consequence of breakdown of the labeled proteins herein. Under the assumption that the protein pool size is not changing, the rate of disappearance of labeled proteins is an estimate of the protein FBR (Holm et al. 2013).

The proteins isolated for assessment of FSR and FBR approaches are similar, assuming the same protocol is used, and therefore, a comparison of the kinetic rates as well as knowledge on and a comparison of the inter‐ and intrasubject variation of the two methods are of interest for future applications. Further, fundamental biological differences are apparent between the conditions under which the protein‐bound tracer abundances are changing during the two approaches. While the FSR is based on measuring the momentary (minutes and few hours) increase in abundance of the amino acid tracer during its immediate exposure to the proteins of interest, the FBR is based on the disappearance of protein‐bound tracers, which were incorporated several days and weeks previously. Hence, although the proteins for both FSR and FBR determination are present in the same sample, the pools of proteins being labeled and, hence, the pool of proteins founding the “proteins of interest” in the two approaches are for certain different in age. For protein structures with complex post‐translational processing before being fully matured and functional such as tendon collagen proteins (Canty and Kadler 2002), the difference between FSR and FBR measurements may be even more pronounced. Therefore, the tendon collagen protein is a suitable model to investigate the impact of protein maturation processing on the disappearance of incorporated tracer from a complex protein structure. In the interest of applicability, the extensive (60–80 days) protein prelabeling and precursor de‐labeling period is a practical challenge especially in human studies. It makes it impossible to apply under “acute” conditions and during standardized diseased conditions when more than two months have to pass from planning to actual measurement. In order to overcome this time gap, we investigate whether a *classic* essential amino acid tracer (exemplified by ^15^N‐phenylalanine, having a much higher turnover rate than water (Holm et al. 2014)) can be used for labeling the proteins of interest with the purpose of measuring the FBR in a similar experimental setup as that with the deuterated nonessential amino acid method.

The present study was designed to explore the methods of measuring the protein‐specific FSR and FBR, and the following purposes were defined: (1) to compare measures of the FSR and FBR on the same subjects and assess the inter‐ and intrasubject variation; (2) to determine the FBR of a tendinous collagen tissue that is known to contain protein pools with very distinct protein turnover rates and undergo extensive post‐translational maturation processes; (3) to investigate whether the FBR protocol can be shortened by the use of a classic essential amino acid tracer to prelabel proteins and determine protein FBR; and 4) to explore in vivo essential amino acid recycling issues both in the context of measuring FSR and FBR.

## Method

### Study 1

#### Subject recruitment and ethical approval

Nine healthy and moderately physically active males were recruited through advertisements at the webpage www.forsogsperson.dk. Their characteristics were as follows: age: 22.4 ± 2.1 years (mean ± SD); body mass index (BMI): 23.0 ± 3.0 kg·m^−2^; and lean body mass (LBM): 61.7 ± 8.6 kg, and they were all recruited as being tracer naïve, that is, never having received any stable isotopically labeled compounds. A written informed consent was obtained before they were enrolled in the experiment that was approved by the Copenhagen Ethics Committee (H‐1‐2010‐007) and conformed to the code of the Helsinki Declaration.

In this study, we aimed at determining the reproducibility of the deuterated water‐based FBR measurement described previously (Holm et al. 2013) (Experiment 1) and comparing the values and their reproducibility with the comparable FSR values (Experiment 2), as well as testing whether the prelabeled FBR method can be performed with the use of a classic essential amino acid tracer (here ^15^N‐phenylalanine) (Experiment 3). Part of the data on a subset of the subjects (*N* = 7) are reported in a previous paper (Holm et al. 2014).

**Figure 1:**
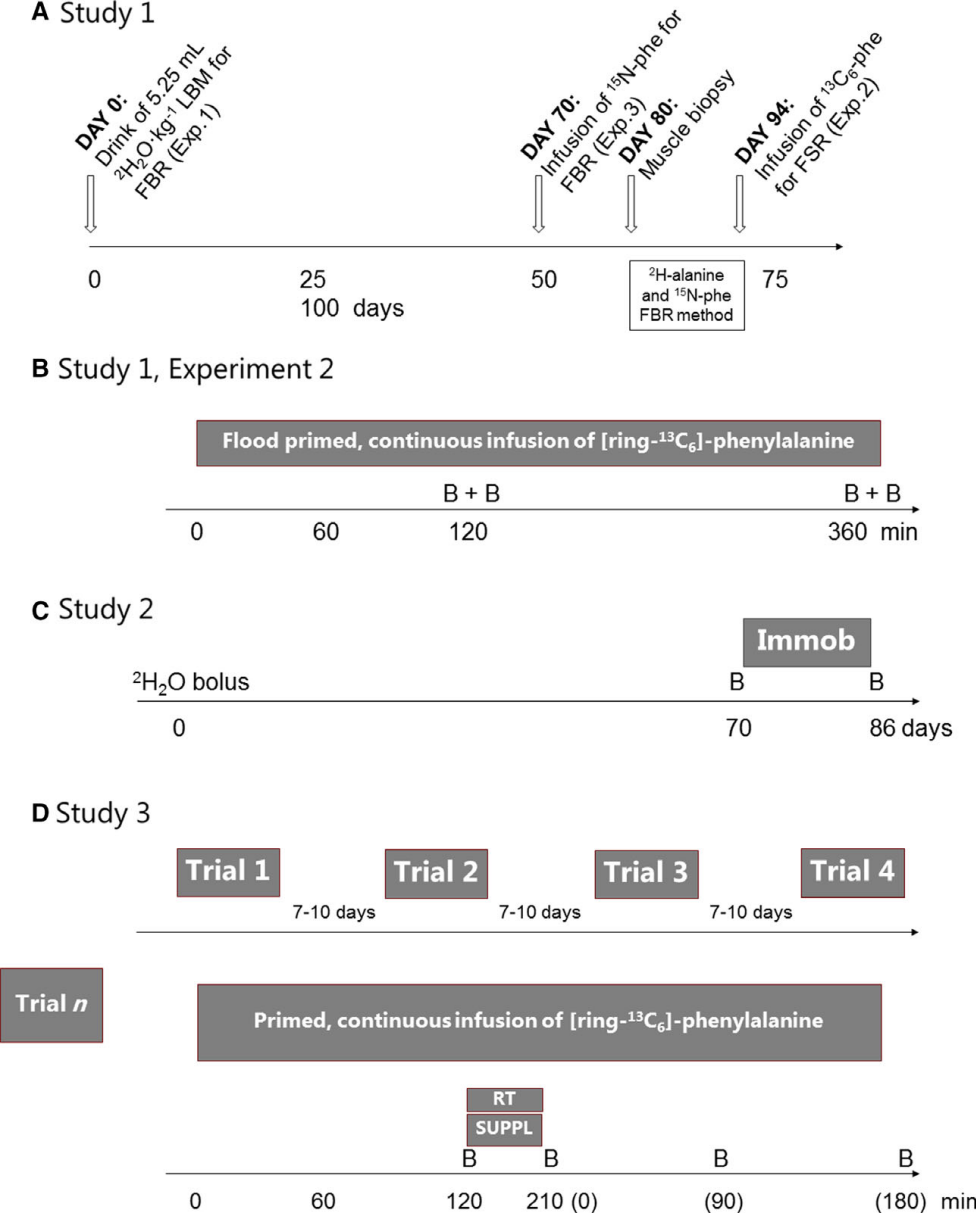
Overview of design of Study 1, experiments 1–3, Study 2, and Study 3. (A) Study 1: The experimental protocol lasted 94 days. Deuterated water was administered on Day 0 as 3–4 boluses over a 2‐h period, and ^15^N‐phenylalanine was infused over 6 h on Day 70, respectively. At days 80 and 94, muscle biopsies were obtained from the vasti lateralis of both legs on study participants to determine the disappearance rate of ^2^H‐alanine and ^15^N‐phenylalanine tracers to calculate the FBR. (B) Study 1, Experiment 2. On Day 94 in Study 1, an infusion of ring‐^13^C_6_‐phenylalanine was given to assess the fractional synthesis rate, FSR, in both legs. (C) Study 2: Determining the incorporation and disappearance of ^2^H‐alanine in patella tendon indispensable protein. Deuterated water was administered on Day 0, and biopsies were obtained from one leg (normally active) at Day 70 and the other leg (after 14 days of immobilization) at Day 86. D) Study 3: Four trials infusing ring‐^13^C_6_‐phenylalanine were conducted separated by 7–10 days and four muscle biopsies obtained in each trial to investigate the incorporation of tracer in muscle proteins. Abbreviations: B: tissue biopsy. LBM: lean body mass. Immob: unilateral cast immobilization. RT: resistance training bout. SUPPL: Leu/branched chain amino acids/essential amino acids/placebo supplement.

The overall design of Study 1 is shown in Figure 1A. Upon inclusion and before starting the experiments, all subjects had their whole‐body lean mass (LBM) determined from a dual‐energy X‐ray absorptiometry scanning (Lunar DPX, Madison, MI; software version 3.6z). At Day 0, subjects drank a total volume of 5.25 mL·kg LBM^−1^ of 99% ^2^H_2_O, which was diluted 1:1 with tap water and provided in 3–4 boluses over a 2‐h period. Blood samples were taken before the deuterated water bolus was given and at days 7, 14, 21, 42, 70, 80, and 94 hereafter to determine the deuterium enrichment of plasma‐free alanine. At Day 70, the subjects arrived to the laboratory in the overnight fasted state, had a venous catheter inserted in the antecubital vein in each arm, had a fasting blood sample drawn, and received a 6‐h primed (32 *μ*mol·kg LBM^−1^) continuous (32 *μ*mol·kg LBM^−1^·kg^−1^) infusion of ^15^N‐phenylalanine. Hereafter, blood samples were taken at 1, 2, 4, and 6 h to determine tracer enrichment. This trial was conducted to label the slow turning over muscle proteins with ^15^N‐pheylalanine, and subjects were therefore also given hourly drinks (0, 1, 2, 3, 4, 5 h) containing 2.5 g L‐leucine and 10 g maltodextrin (both Fagron, GmbH & Co.KG, Barsbüttel, Germany) to enhance protein synthesis improving the incorporation of phenylalanine tracer into skeletal muscle during the infusion trial. The time elapse for tracer infusion prior to FBR assessment was chosen as a trade‐off to allow a high abundance of protein‐bound tracer and estimated adequate time to achieve negligible levels of free tracer in the blood and intramyocellular pools also taking the recycling from fast turning over proteins into account (Kaufman 1999). At Day 80, the subjects reported to the laboratory and had a muscle biopsy taken from each leg using standard procedures under local anesthesia (lidocaine 1%) aiming at determining the muscle protein‐bound abundances of ^2^H‐alanine and ^15^N‐phenylalanine and venous blood sample to determine the enrichments of the same two tracers in the plasma. Finally, at Day 94, the subjects reported to the laboratory to have another muscle biopsy taken in order to assess the disappearance rate of ^2^H‐alanine and ^15^N‐phenylalanine. In addition, on Day 94, a 6‐h infusion trial was conducted. Therefore, subjects were instructed not to perform any strenuous exercise on 3 days prior to Day 94 and they arrived to the laboratory in the morning by car or public transportation and were in the overnight fasted state. After placing two catheters in the antecubital veins of each arm and drawing a basal blood sample, the flood‐prime was given as a total of 1665 mg phenylalanine: 1480 mg unlabeled (8.97 mmol) and 185 mg labeled [ring‐^13^C_6_]‐phenylalanine (1.08 mmol) followed by a continuous infusion (8 *μ*mol·kg LBM^−1^·h^−1^) of [ring‐^13^C_6_]‐phenylalanine. Bilateral vastus lateralis muscle biopsies were obtained after 120 and 360 min, and blood samples were collected throughout the trial. Using the change in incorporation of phenylalanine, between 120 and 360 min, we determined the myofibrillar protein synthesis rate in the vastus lateralis muscles from both legs (Fig. 1B).

From the bilateral muscle biopsies obtained at days 80 and 94, we analyzed samples for abundances of [^2^H]‐alanine and [^15^N]‐phenylalanine required for calculating the FBR of myofibrillar proteins based on the two tracer administration approaches. Further, from samples obtained on Day 94 during the ring‐^13^C_6_‐phenylalanine tracer experiment, we analyzed samples from subjects in the basal, overnight fasted state to calculate the myofibrillar protein FSR using the precursor–product approach.

### Study 2

#### Subject recruitment and ethical approval

Thirteen healthy and sedentary elderly males (age: 68.8 ± 6.5 years [mean ± SD]; body mass index [BMI]: 24.9 ± 1.8 kg·m^−2^, lean body mass [LBM]: 57.2 ± 4.7 kg) were recruited through newspaper advertisements. They were all recruited as being tracer naïve, and the written informed consent was obtained before subjects were enrolled in the experiment that were approved by the Copenhagen Ethics Committee (H‐1‐2010‐007) and conformed to the code of the Helsinki Declaration.

Subjects were enrolled in an experiment aiming at determining the tendon connective tissue protein as well as the muscle protein FSR before and after a 2‐week period of lower limb immobilization (Dideriksen et al. 2016). Further, deuterated water was provided to the subjects 70 days prior to the immobilization‐retraining protocol to determine the myofibrillar protein FBR over the period of immobilization as well as during the retraining period (Dideriksen et al., unpublished). Upon inclusion, subjects had their whole‐body lean mass (LBM) determined from a dual‐energy X‐ray absorptiometry scanning (Lunar DPX, Madison, MI; software version 3.6z). At Day 0, subjects drank a total volume of 5.25 mL·kg LBM^−1^ of 99% ^2^H_2_O, which was diluted 1:1 with tap water and provided in 2–3 boluses over a 1‐h period. At days 70 and 86, the subjects arrived at the laboratory by car or public transportation after an overnight fast and had a patella tendon biopsy taken using a disposable 14‐G needle (Bard Magnum Biopsy Instrument, C.R. Bard Inc., Covington, Kentucky) under local anesthesia (lidocaine 1%). The nonimmobilized leg was biopsied at Day 70 and the immobilized leg at Day 86 during an amino acid tracer infusion protocol to assess the fractional synthesis rate of tendon connective tissue proteins (Dideriksen et al. 2017). Hence, the tendon samples prepared for amino acid tracer abundance were also analyzed for protein‐bound abundances of ^2^H‐alanine. On both days, subjects were instructed to refrain from alcohol and strenuous physical activity and to follow their normal eating pattern 72 h prior to the tendon biopsy sampling. Furthermore, no intake of caffeine was allowed 24 h before the tendon biopsy sampling.

### Study 3

#### Subject recruitment and ethical approval

Eight healthy males who were accustomed to strength training (age: 26.5 ± 65.6 years [mean ± SD]; body mass: 83.5 ± 8.3 kg, height: 180.5 ± 8.4 cm) were recruited through adverts at local gyms and at www.gih.se. They were all recruited as being tracer naïve, and the written informed consent was obtained before subjects were enrolled in the experiment that were approved by the Regional Ethical Review Board in Stockholm (Dnr 2014/96‐31/2) and conformed to the code of the Helsinki Declaration.

The main purpose of this study was to determine muscle protein FSR during 3‐h recovery following leg press exercise and ingestion of leucine, all three branched‐chain amino acid, a mixture of essential amino acids, or flavored water acting as a placebo supplement (Moberg et al. 2016). The supplements were given to the subjects in a counter‐balanced order, and the trials were separated by 7–10 days. The FSR of muscle protein was measured using L‐ring‐^13^C_6_‐phenylalanine infusion to determine FSR following resistance exercise. Prior to the series of four trials, all subjects had gone through medical screening and maximal strength testing and completed three sessions of heavy leg press exercise for training customization.

On the day of the trials, subjects reported to the laboratory at 6 am following an overnight fast. Subjects were instructed to refrain from all types of vigorous physical activity and to eat the same diet during the 2 days prior to the experiments. Upon arrival, subjects were instructed to lie down for insertion of venous catheters in the antecubital vein of both arms. After a baseline blood sample was drawn, a primed constant infusion of L‐ring‐^13^C_6_‐phenylalanine (prime 2 *μ*mol·kg^−1^; 0.05 *μ*mol·kg^−1^·min^−1^, Cambridge Isotope Laboratories, Danvers, MA) was initiated and continued thereafter for the duration of the experiment (~6 h). A first *vastus lateralis* muscle biopsy was collected after two hours of tracer infusion, at rest, using a Weil‐Blakesley conchotome (AB Wisex, Mölndal, Sweden) under local anesthesia (Henriksson 1979). Following the baseline muscle biopsy, subjects performed a session of heavy leg press exercise that lasted approximately 50 min. The amino acid supplements were consumed during as well as after exercise and administered in a randomized order. Immediately after exercise (time (0) zero at Fig. 1D), a second muscle biopsy was collected, followed by a third and fourth after 90 and 180 min of recovery, respectively. All biopsies were taken from alternating legs (i.e., 1st; right, 2nd; left, 3rd; right, 4th; left) all from a new incision approximately 2 cm proximal to the previous one. During the second trial, the biopsies were collected 2 cm medial to the previous ones. In the third and fourth trials, the biopsies were collected in the same manner but 2 cm proximal to the previous ones. The muscle tissue was rapidly blotted free from blood, frozen in liquid nitrogen, and subsequently stored in −80°C. Muscle samples were then lyophilized and meticulously dissected clean from blood and connective tissue under a light microscope, leaving only very small intact bundles of fibers that were mixed to obtain a highly homogenous sample free of contaminating tissue

#### Analyses

Plasma phenylalanine tracer enrichment and concentrations and alanine tracer enrichments were determined by using 200 *μ*L plasma, added a known amount of [U‐^13^C_9_]‐phenylalanine for use as an internal standard, acidified with 1 mL 50% acetic acid, and poured over resin columns preconditioned with 1 mL 50% acetic acid. After five washes with Millipore water two times, 1 mL NH_4_OH was added to the resin columns and the eluent collected. After being dried down under a stream of nitrogen, the purified amino acids were derivatized using MtBSTFA + 1% tBDMCS (Regis Technologies, Inc., Morton Grove, IL) and acetonitrile, volume relation 1:1. The tBDMS derivatives of the amino acids were analyzed by tandem mass spectrometry using a Thermo Scientific, TSQ Quantum GC‐MS/MS (San Jose, CA), under electron ionization mode. The derivatives were separated on a 30 m CP Sil‐8 CB capillary column (ChromPack, Varian, Palo Alto, CA) using PTV mode injection of 1 *μ*L. The ^13^C and ^15^N enrichments of the phenylalanine and ^2^H‐alanine tracers were measured by GC‐MS/MS by monitoring the fragments of the [M‐COOtBDMS]^+^ parent ions of m/z [234–243] and [158–159] for phenylalanine and alanine, respectively, in the neutral loss mode [‐56].

Muscle specimens from Study 1 of ~20 mg wet weight were homogenized (Fastprep, 120A‐230, Thermo Savant, Holbrook, NY, USA) for 2 × 45 sec in 1.5 mL ice‐cold Millipore saline water. After a spin (5500 g, 10 min, 4°C), the supernatant was transferred to new cooled vials containing 1.5 mL 100% acetic acid, vortexed, and poured over resin columns preconditioned with 1 mL 50% acetic acid. The amino acids were then purified over resin columns and derivatized as their tBDMS derivatives as described for the plasma free amino acids. The myofibrillar protein fraction was isolated from the remaining protein pellet by adding 1 mL of a homogenization buffer (Tris 0.02 mol·L^−1^ [pH 7.4], NaCl 0.15 mol·L^−1^, EDTA 2 mmol·L^−1^, Triton‐X 100 0.5%, sucrose 0.25 mol·L^−1^), homogenizing 2 × 45 sec and following the procedures reported previously (Holm et al. 2014). The myofibrillar protein pellet was hydrolyzed overnight in 1 ml of 6 mol·L^−1^ HCl at 110°C. The liberated amino acids were purified over cation exchange resin columns and derivatized as their N‐acetyl‐n‐propyl (NAP) esters for determination of the ^2^H‐alanine and ^13^C‐ and ^15^N‐phenylalanine abundances.

Tendon specimens obtained in Study 2 were prepared as previously described (Dideriksen et al. 2013). Briefly, connective tissue collagen protein was isolated from tendon biopsies by homogenization first 5 × 15 s in 1 mL of homogenization buffer (Tris 0.02 mol·L^−1^, pH 7.4, 0.15 mol·L^−1^ NaCl, 2 mmol·L^−1^ EDTA, and 0.05% Triton‐X 100), left for 2 h, and spun (1600 g, 20 min, 4°C). Hereafter, 1 mL of high‐salt solution (0.7 mol·L^−1^ KCl) was added to the pellet, which was left overnight at 4°C. After a spin (1600 g, 20 min, 4°C), 1 mL of KCl was added to the pellet, which was homogenized and left for 2 h. After spinning (1600 g, 20 min, 4°C), the pellet was washed once in 70% ethanol and hydrolyzed overnight in 1 ml of 6 mol·L^−1^ HCl at 110°C. The liberated amino acids were purified over cation exchange resin columns and derivatized as their N‐acetyl‐n‐propyl (NAP) esters to determine the abundance of ^2^H‐alanine.

In Study 3, the myofibrillar protein tracer enrichments were determined from 10 mg of lyophilized muscle tissue. Homogenization was performed in ice‐cold buffer (2 mmol·L^−1^ HEPES (pH 7.4), 1 mmol·L^−1^ EDTA, 5 mmol·L^−1^ EGTA, 10 mmol·L^−1^ MgCl_2_, 50 mmol·L^−1^ *β*‐glycerophosphate, 1% TritonX‐100, 1 mmol·L^−1^ Na_3_VO_4_, 2 mmol·L^−1^ dithiothreitol, 1% phosphatase inhibitor cocktail [Sigma P‐2850], and 1% [w/w] Halt Protease Inhibitor Cocktail [Thermo Scientific, Rockford]) utilizing a BulletBlender (NextAdvance, New York). The homogenates obtained were rotated for 30 min at 4°C and subsequently centrifuged at 10,000*g* for 10 min at 4°C. The resulting pellet was collected and washed once with 500 *μ*L purified H_2_O, then dissolved in 1 ml homogenization buffer (see above), and centrifuged at 1600*g* for 20 min at 4°C. The supernatant was discarded, and the pellet containing myofibrillar protein was lyophilized prior to hydrolysis.

All NAP‐derivatized samples were analyzed on the IRMS equipment. For ^13^C and ^15^N abundances in phenylalanine, the GC‐combustion system was used as described previously (Holm et al. 2014). The ^2^H‐alanine abundances were determined on the GC‐temperature conversion setting following previously described procedures (Holm et al. 2013).

#### Calculations

All enrichments were determined as the tracer‐to‐tracee ratio by subtracting the isotope ratio of a background sample from the isotope ratio of the labeled samples.

The protein fractional synthesis rate was determined by using the precursor–product approach: FSR = ∆protein‐bound tracer × E_precursor_^−1^ × incorporation time^−1^. The myofibrillar protein‐bound tracer abundance over the course of a 4‐h period (120–360 min in study 1 and 2) or a 3‐h period (0–180 min, study 3) was determined. The muscle free tracer enrichment was used as a surrogate measure of the precursor enrichment.

The protein fractional breakdown rate was determined using the procedure described previously (Holm et al. 2013). Basically, loss of ^2^H‐alanine over time from two protein samples was as estimate of the rate of protein degradation. It was determined by log transforming the measured enrichments of protein‐bound tracer at two protein samples and the slope of the line made by the two samples were the rate constant.

#### Statistics

When more than two sampling points are reported within one group, a one‐way ANOVA with repeated measures was used, whereas a two‐way ANOVA was applied to compare multiple sampling points in two or more groups. The myofibrillar FSR and FBR values from left and right legs were set up assuming sphericity, whereas tendon and myofibrillar enrichments over prolonged period of time were set up not to assume sphericity. When significant overall effects, post hoc Holm–Sidak tests were performed, and when appropriate, t‐tests were applied to compare only two data points. The statistical software Prism 7.04 (GraphPad, CA, USA) was used for all statistical tests. Data are reported as mean ± standard error of mean (unless otherwise stated), and a *P*‐level below 0.05 was considered as significant.

## Results

### Study 1, Experiment 1: Plasma‐free and muscle protein‐bound ^2^H‐alanine enrichment

**Figure 2:**
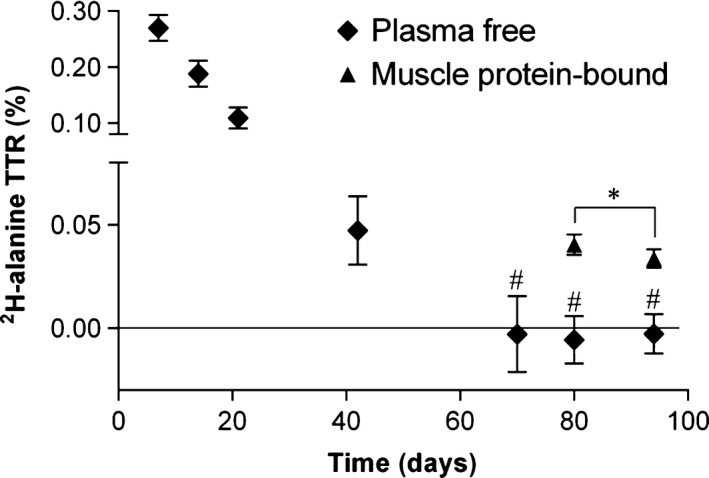
Experiment 1, administration of ^2^H_2_O and the subsequent enrichment of ^2^H‐alanine. After intake of deuterated water on Day 0, the deuterium atoms are transferred to numerous metabolites and alanine receives up to four enzyme‐dependent deuterium atoms allowing high analytical sensitivity on amino acid enrichment. At days 7, 14, 21, 42, 70, 80, and 94, plasma samples were obtained and symbol ♦ shows the enrichments. # denotes plasma enrichments not different from zero. At days 80 and 94, muscle biopsies were obtained bilaterally and symbol ▲ shows myofibrillar protein‐bound ^2^H‐alanine as a mean from both legs from all subjects at the two separate days. * denotes significantly different enrichment in muscle protein‐bound ^2^H‐alanine. Symbols are mean and bars SEM.

Intake of ^2^H_2_O resulted in an ^2^H‐alanine enrichment in plasma at Day 7 of 0.27 ± 0.02%, which decreased with a rate of 6.5 ± 0.9%·d^−1^ to an enrichment not significantly different from zero at days 70, 80, and 94 (Fig. 2, symbol ♦ marked with #). Myofibrillar protein‐bound ^2^H‐alanine enrichment as a mean of the two legs was 0.040 ± 0.005% and 0.034 ± 0.004% at days 80 and 94, respectively (Fig. 2, symbol ▲), and the intra‐individual variation (difference between the measures in each leg related to the mean of the samples taken concomitant from the vastus lateralis from each leg) at days 80 and 94 was 2.2 ± 4.1% and 1.6 ± 2.6%, respectively.

### Study 1, Experiments 1 and 2: Myofibrillar protein turnover rates

**Figure 3:**
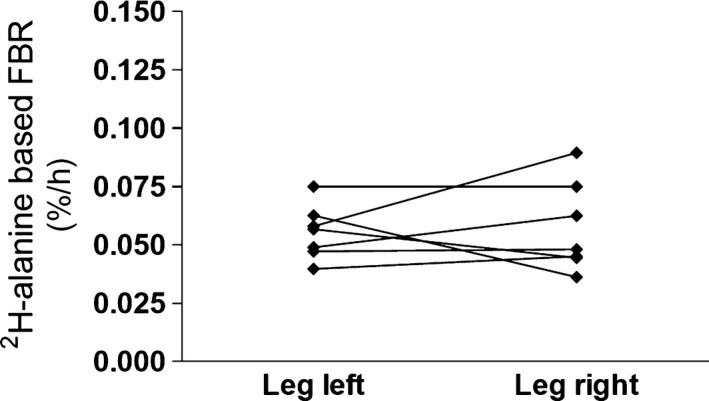
Experiment 1, administration of ^2^H_2_O and the measurement of ^2^H‐alanine‐based myofibrillar protein fractional breakdown rate. Based on symbols ▲ on Figure 2, the FBR was determined as an average over a 14‐day period from Day 80 to Day 94 after intake of deuterated water. For each of the vastus lateralis muscles in both legs, the FBR was determined of each subject and plotted as the left and right leg connected with a line (*P* = 0.81).

After intake of deuterated water on Day 0, the myofibrillar protein FBR in vasti lateralis muscles as a gross mean from Day 80 to Day 94 was calculated to 0.056 ± 0.004%·h^−1^ and 0.057 ± 0.007%·h^−1^ for left and right legs, respectively (Fig. 3, *P* = 0.81). The intra‐individual variation was 0.5 ± 12.0% (0.09–53.5%) (calculated as the difference between paired measurements divided by the mean of the two measurements), and interindividual coefficient of variation of the mean between the legs was 22%.

**Figure 4:**
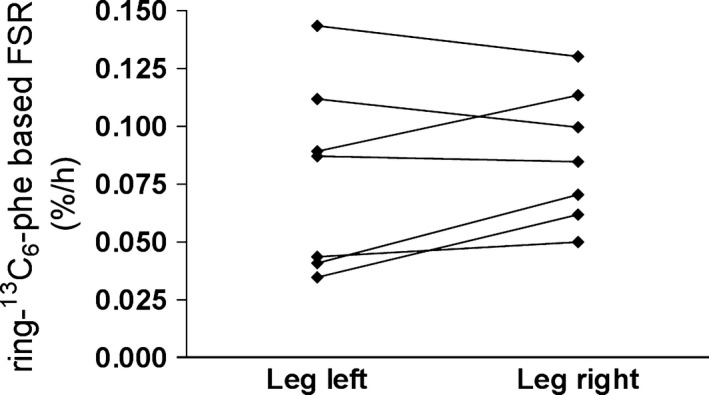
Experiment 2, infusion of ring‐^13^C_6_‐phenylalanine and the measurement of the myofibrillar protein fractional synthesis rate. Based on the rate of incorporation of ring‐^13^C_6_‐phenylalanine over a 4‐ to 5‐h infusion trial on Day 94, the myofibrillar protein fractional synthesis rates were calculated in each of the vastus lateralis muscles in the left and right legs of each subject and paired values are connected with a line (*P* = 0.27).

For comparison, we determined, in Experiment 2, the overnight fasted and resting myofibrillar protein fractional synthesis rates (FSRs) also in the vasti lateralis muscles of both legs and it was 0.079 ± 0.015%·h^−1^ and 0.087 ± 0.011%·h^−1^ for left and right legs, respectively (Fig. 4, *P* = 0.27). The intra‐individual variation was 17.5 ± 10.7% (2.8–56.2%), and the interindividual coefficient of variation of the mean between the legs was 43%.

**Figure 5:**
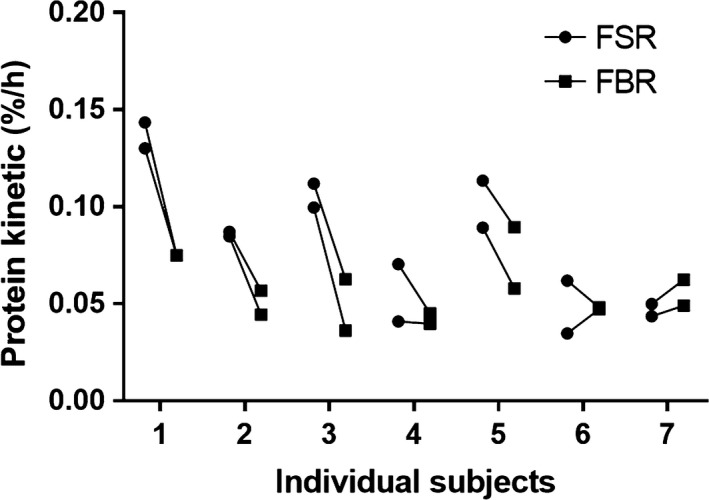
Experiments 1 and 2: comparison of the myofibrillar FSR and FBR values. Paired values of myofibrillar FSR and FBR for each of the research participants are connected with a line and appear pairwise, one set of values for each leg obtained at the same time. A two‐way ANOVA test reveal difference between FSR and FBR values (*P* < 0.01), with a 4‐h resting and fasting myofibrillar FSR being 1.48 ± 0.19 fold higher than the comparable 14‐d gross mean of myofibrillar FBR.

A comparison of the two protein turnover kinetics revealed that the FSRs in average were 1.48 ± 0.19 fold higher than the paired FBR rates (Fig. 5, *P* < 0.01).

### Study 2: Patella tendon connective tissue protein ^2^H‐alanine enrichment

**Figure 6:**
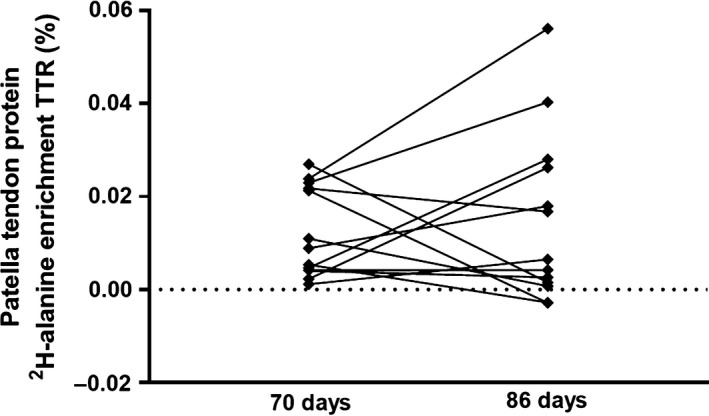
Study 2: administration of ^2^H_2_O and ^2^H‐alanine enrichments in patella tendon tissue proteins after 70 and 86 days. Deuterated water was provided a group of elderly males enrolled in a clinical trial investigating skeletal muscle and tendon tissue adaptation to immobilization. Patella tendon biopsies were obtained from one leg at Day 70 and from the contralateral leg at Day 86 (after 14 days of immobilization). ^2^H‐alanine enrichments from each leg are depicted, and within subjects, enrichments are connected with a line (*P* = 0.55).

In Study 2, another group of participants had a patella tendon biopsy taken from one leg at Day 70 and the other leg at Day 86; after this, latter leg had been immobilized for 14 days. The ^2^H‐alanine enrichments in the patella tendon connective tissue protein is depicted in Figure 6. There was a significant (*P* < 0.05) enrichment (>zero) at both days 70 and 86, however, with no difference between the 2 days (*P* = 0.55). Since the enrichments were not different at the 2 days, we calculated the intra‐individual variation as the difference between the enrichments divided by the mean enrichment to be 50.0 ± 60.4% (1–622%) and the interindividual coefficient of variation at days 70 and 86 of 79% and 121%, respectively.

### Study 1, Experiment 3: Plasma‐free and muscle protein‐bound ^15^N‐phenylalanine

**Figure 7:**
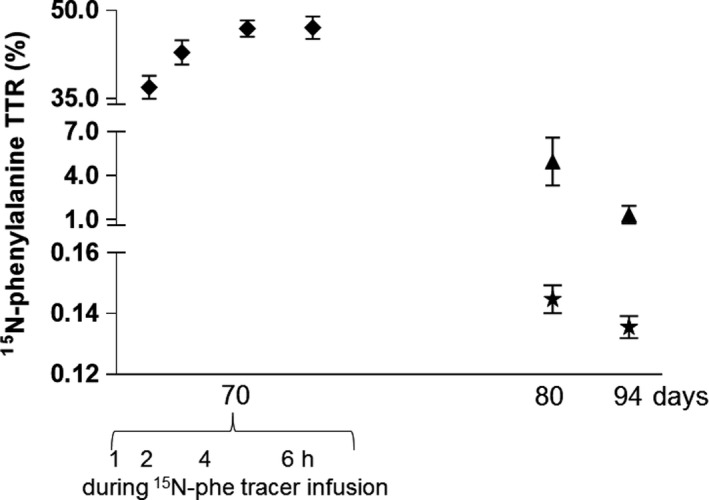
Experiment 3, infusion of ^15^N‐phenylalanine and enrichment in plasma and in skeletal muscle. On Day 70, subjects in Study 1 received a 6‐h continuous infusion containing a high amount of ^15^N‐phenylalanine and plasma enrichments are shown after 1, 2, 4, and 6 h of infusion (symbol ♦). The subjects went home after the 6‐h infusion at Day 70 and came in again at days 80 and 94 (i.e., 10 and 24 days after the infusion) and had a blood sample drawn and muscle biopsies taken (same as for Experiment 1). Symbol ▲ denotes plasma ^15^N‐phenylalanine enrichments at days 80 and 94, and symbol ★ denotes means of myofibrillar protein‐bound ^15^N‐phenylalanine enrichment at days 80 and 94. Symbols are mean and bars SEM.

In Study 1, the subjects were given an infusion of ^15^N‐phenylalanine at Day 70, which resulted in a ^15^N‐phenylalanine enrichment in plasma of 36.9 ± 2.0% and 47.1 ± 1.9% at 1 and 6 h, respectively (Fig. 7, symbols ♦), which decreased to 4.97 ± 1.63% and 1.36 ± 0.60% at days 80 and 94, respectively (Fig. 7, symbol ▲). The mean (one muscle biopsy from each leg at each time point) muscle myofibrillar protein‐bound ^15^N‐phenylalanine enrichment was 0.145 ± 0.005% and 0.136 ± 0.004% at days 80 and 94, respectively (*P* < 0.05) (Fig. 7, symbol ★).

### Study 3: Increasing abundance of ring‐^13^C_6_‐phenylalanine in myofibrillar protein with repeated infusions

**Figure 8:**
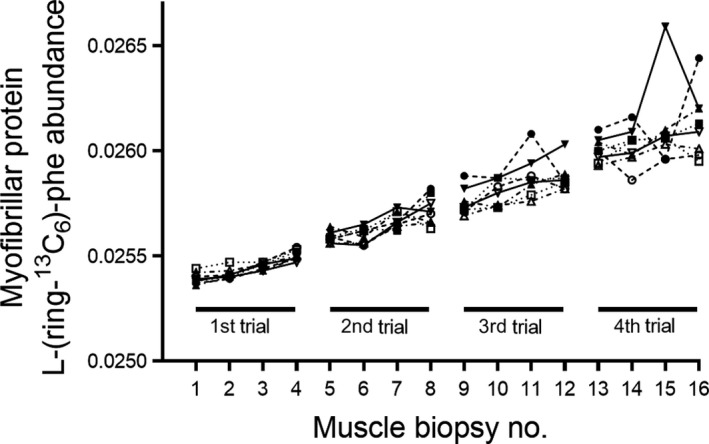
Individual values of L‐[ring‐^13^C_6_]‐phenylalanine abundance in the myofibrillar protein fraction and calculated FSR in four trials on the same subjects. (A) The data on the Y‐axis is ^13^C_6_‐phenylalanine tracer abundance measured by GC‐C‐IRMS in 16 biopsies from each of the eight subjects. Biopsy numbers 1, 5, 9, and 13 were taken fasted at rest in the morning after a primed 2‐h infusion period; biopsy numbers 2, 6, 10, and 14 were taken immediately after leg press exercise and supplement intake approximately 60 min after the previous one (defining time zero); biopsy numbers 3, 7, 11, and 15 were taken after 90 min of recovery and biopsy numbers 4, 8, 12, and 16 after 180 min of recovery. (B) Myofibrillar protein FSR during the 3‐h postexercise/supplement period in the four trials, that is, calculated between 0 (zero)‐ and 180‐min biopsies. The intracellular ^13^C_6_‐ phenylalanine enrichment was used as precursor pool in the calculations. Each trial was separated by 7–10 days.

The myofibrillar protein tracer enrichment in the 16 muscle biopsies collected during the four separate trials for all subjects is depicted in Figure 8. During the first trial, there is an increase throughout the experiment and the individual variation is relatively small. However, during the second, third, and fourth trials, the variation in enrichment increases progressively and, in some cases, this results in negative delta values and hence negative FSR values. Particularly during the third and fourth trials, both very large delta increases and decreases were noted over time, particularly in the latter. The mean delta tracer‐to‐tracee ratio for all biopsies in the different trials was 0.0004 ± 0.00019 (mean ± SD) for the first, 0.0004 ± 0.00068 for the second, 0.0004 ± 0.00082 for the third, and 0.0004 ± 0.00180 for the fourth, noticeably illustrating the increase in variation in tracer enrichment with repeated trials.

## Discussion

In this study, we started by comparing the variations of the two methods to determine the protein‐specific turnover rates in skeletal muscle, namely the disappearance rate of protein‐bound deuterated alanine as an estimate of the fractional breakdown rate (FBR) versus the direct incorporation rate of carbon‐13 phenylalanine in the similar protein pool.

We found no statistical difference between the legs for any of the two rates and the intrasubject variation (left and right legs) was within the same range: 0.09–53.5% for FBR and 2.8–56.2% for FSR. We do neither ascribe activity‐related side‐specific differences nor different fiber‐type compositions (Mittendorfer et al. 2005) to be causing the surprisingly large intrasubject variation. The included participants were recruited as being recreationally active with no specific preferences for physical activities or sports. Also, laboratory processing of muscle tissue and analysis cannot have caused the largest observed variation. Therefore, we suggest that the variation between legs must be caused by actual local intramuscular biological/physiological differences; for example, distinct anatomical locations within the muscle have different protein turnover rates, maybe due to differences in recruitment pattern; and different areas within the muscle have different blood perfusion.

The between‐subject coefficient of variation of the FBR measurement of 22% was somewhat smaller than that of the FSR measurement, 43%. The immediate interpretation of this difference is that the synthesis rates are subject to larger fluctuations than the breakdown rates (Phillips et al. 2009) although it lacks evidence. In support of this notion, the conditions around the FSR trial (measured over minutes to hours) are easier to control and standardize in opposed to the FBR, which is an average over days. We cannot reject that a more thorough standardization prior to the FSR measurement period potentially could decrease the interindividual variation for this measure, though would require that research participant met into the laboratory one or more days prior to the experiment.

Of relevance to discuss is also the numerical difference between the FBR and FSRs, with FBR 14d gross average FBR being 77 ± 10% lower than the acute 6‐h average FSR in the resting, fasting condition, which would be expected to be an underestimation of the gross daily average synthesis because of the absence of muscle activity and nutrients (Holm et al. 2010). Further, had the participants been heavily physically active like those in Study 3, the numerical turnover rates and the differences between FSR and FBR might have been different. Further, we isolate the same myofibrillar proteins, but the proteins with tracer bound in the prelabeling FBR protocol and with tracer incorporated during the immediate exposure in the FSR protocol may diverge in type, age, and maturation stage and hence diverge in turnover rate. The numerical deviation between the two rates must therefore be ascribed to the methodological differences rather than physiology. Hence, we argue that the FBR and FSR are not numerically directly comparable and must not be used to extrapolate to the net balance.

**Figure 9:**
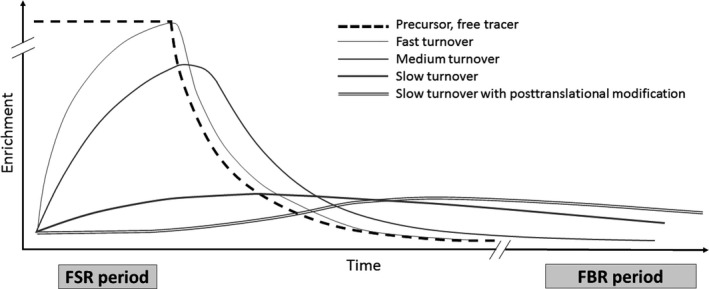
Theoretical illustration of tracer enrichments in different pools of proteins during the FSR and FBR measurement periods. The hatched line is the free amino acid tracer enrichment in the precursor pool. The slim line represents the enrichment of the protein‐bound tracer in fast turning over proteins, and the medium thick and thick lines represent enrichments of protein‐bound tracers in medium and slow turning over proteins. The double line is the pool of a slow turning over protein after it has been post‐translationally modified, why it takes some time before the label starts to appear herein. The FSR is a gross mean of the weighted average made up of the turnover rate and the relative abundance of a protein in the pool. Tracee amino acids from slow turning over proteins will dilute the isotope ratio measurement and decrease the enrichment. At the time of FBR determination, all fast turning over proteins have lost their label and only proteins with slow turnover will still be labeled. Hence, the FBR determination may be a measure of proteins with a more homogenous turnover rate.

We continued our exploration of tracer data originating from a protein pool undergoing a complex post‐translational maturation process (exemplified by human tendinous tissue). Some proteins are synthesized as preproteins, from which pool only a fraction is further processed and matured into a functional matrix protein – for example, the collagenous proteins (Babraj et al. 2002; Bechshoft et al. 2017). When exposed acutely to tracer, which is the condition in the FSR protocol, fast turning over proteins within the isolated protein pool will incorporate tracer quickly and will increase the gross tracer abundance in the protein fraction above that of what slower turning over proteins contribute with. This we illustrate by reporting the abundance of ^13^C‐labeled proline and hydroxyproline in a pool of tendon proteins 2 h after a flood of ^13^C‐proline in two studies from our research group ((Hansen et al. 2009; Petersen et al. 2011), Table 1). While a marked increase in ^13^C‐proline appeared in the mixed tendinous proteins within the 2‐h protocol, no label appeared on the hydroxyproline residues, which have the ^13^C‐proline as precursor but require time for the post‐translational processing of the precollagen protein. In contrast, the labeling of these high turning over proteins will be absent in the prelabeling FBR protocol. Due to their high turnover rate, these proteins will degrade rather quickly. This means that once the precursor enrichment has dropped to zero, these proteins will be degraded and no longer contribute with label to the pool of proteins at the time of FBR determination. Further, even if the protein(s) of interest undergoes a complex post‐translational modification process, the situation will be the same. To investigate the impact of this phenomenon on the FBR assessment, we obtained patella tendon biopsies over the course of a 16‐day period 70 days after intake of deuterated water and found similar tracer enrichments at 70 and 86 days albeit with a huge variation (Fig. 6). The turnover of structural collagenous tissue is debated as acute measures of synthesis reveal rather high turnover rates (e.g., Holm et al. 2014; Miller et al. 2005, 2007), whereas other prelabeling approaches reveal almost no turnover in adult life (Heinemeier et al. 2013; Bechshoft et al. 2017). Obviously, these data cannot both be true for the same proteins. However, they most likely demonstrate the existence of different pools of connective tissue proteins that are present in the isolated protein pool, which is supported by animal work (Bechshoft et al. 2017). In Figure 9, we illustrate the enrichment in pools of proteins with different turnover rates and maturation processes as a function of time. FSR and FBR values of tissues/protein fractions with such complex nature should be interpreted with caution, and the absolute values cannot be directly compared. Again, one should be reminded that these data just exemplify a methodological challenge, while the numerical data are less important and may diverge dependent of participant characteristic (age, physical activity level, health state, etc.) and investigated condition (fasting, activity level/immobilized, health state, etc.).

**Table 1:** Abundance of ^13^C‐labeled proline and hydroxyproline in skin and patella tendon proteins

	Skin protein	Patella tendon protein		Ref.
	BGrd	REST	EXC	
Proline	1.0870 ± 0.0015	1.0887 ± 0.0018^a^	1.0886 ± 0.0019^a^	Hansen et al. (2009)
Hydroxyproline	1.0810 ± 0.0034	1.0813 ± 0.0036	1.0812 ± 0.0034	
		Gross average		
Proline	1.0890 ± 0.0003	1.0928 ± 0.0050		Petersen et al. (2011)
Hydroxyproline	1.0858 ± 0.0003	1.0871 ± 0.0060		

From two studies (Hansen et al. 2009; Petersen et al. 2011), we present the abundances of ^13^C‐proline and ^13^C‐hydroxyproline in skin protein from background samples of tracer virgins and in patella tendon protein 2 h after a flood with ^13^C‐proline. The proline abundances were significantly elevated in the patella tendon protein in both studies, top: two‐way ANOVA, *P* = 0.0003, and *a* denotes different from background, *P* < 0.01. Bottom: paired *t*‐test, *P* = 0.003, whereas the abundances of ^13^C‐hydroxyproline did not change as a result of the ^13^C‐proline flood: top: two‐way ANOVA, *P* = 0.58 and bottom: paired *t*‐test, *P* = 0.32. Values are mean ± SD.

The third objective of the study was to investigate the feasibility of using a classic essential amino acid tracer to prelabel proteins for the determination of FBR with the main purpose to shorten the protocol and make it more applicable. To study this, we exposed subjects in Study 1, Experiment 3, with ^15^N‐phenylalanine tracer 10 days prior to the 14‐day FBR measurement period. This protocol was based on a compromise between (1) least time from tracer exposure to FBR measurement with the purpose to shorten the experimental protocol and to obtain as high analytical sensitivity as possible of the tracer enrichments in the proteins of interest and (2) enough time to allow the free tracer (phenylalanine) to disappear based on the natural flux rates. Phenylalanine has a quick clearance rate in human circulation (Holm et al. 2014), half‐life estimated in the range 60–120 min (Kaufman 1999). Therefore, 10 days should in theory be sufficient time to allow infused free phenylalanine tracer to disappear from the watery pools, become metabolized, and/or incorporated into proteins. The rather high enrichment of free phenylalanine tracer in the blood after 10 days (4% at Day 80) and 24 days (1% at Day 94) (Fig. 7) reveals a marked recycling. An equilibrium of tracer abundances between extra‐ and intracellular pools must be assumed at these points in time, and hence, a continuous incorporation of tracer will take place at the time of FBR measurement, between days 80 and 94. The crucial assumption of null precursor enrichment during the FBR measurement period is thereby violated, and therefore, the FBR cannot be determined in this experimental setting used here. To investigate whether recycling would be an issue, we looked into some unpublished data (Table 2 and Fig. 8). From eight tracer naive individuals, we obtained tendon and skin biopsies before tracer exposure, and from tendon again after 2 h after a flood with ^13^C‐proline, and again 40 days after the flood. The connective tissue pellet was isolated, and the abundance of ^13^C‐proline was determined (Petersen et al. 2011). The results reveal that ^13^C‐proline is highly present at 2 h and 40 days after a flood (Table 2A). Similarly, in muscle biopsies from an ongoing study (Bechshoeft et al. 2016), we determined the ^13^C‐phenylalanine abundances in the myofibrillar protein fraction 12 month after a 6.5‐h primed, continuous infusion of L‐ring‐^13^C_6_‐phenylalanine and found that 33% of the tracer abundance found at the end of the infusion trial was still present 12 month later (Table 2B). In both situations, the essential amino acid tracer abundance in the protein fractions is most likely a combination of the presence of matured and functional matrix proteins as well as newly synthesized proteins due to intracellular recycling of the essential amino acid tracer. Based on these data, however, we conclude that it is very unlikely that it will be possible to use phenylalanine tracer to determine the FBR using the prelabeling approach.

**Table 2:** Abundance of ^13^C‐labeled amino acid in tissue proteins. (A) Abundance of ^13^C‐proline in patella tendon and skin extracellular connective tissue protein in tracer virgins and 2 h after a flood of tracer and 40 days after the flood (unpublished data). For the patella tendon protein, a one‐way ANOVA with repeated measures, not assuming sphericity, revealed a *P*‐value of 0.049, which was obtained by the differences between background and the two postflood time points. For the skin protein, a *t*‐test showed no difference, *P* = 0.85. (B) Abundance of ^13^C‐phenylalanine in myofibrillar proteins in tracer virgins exposed to a primed, continuous infusion of L‐ring‐^13^C_6_‐phenylalanine before and after a 12‐month intervention period (subgroup from the study described in Bechshoeft et al. (2016)). The tracer abundance was different at all time points (*P* < 0.0001), (A) denotes different from 90 min infusion PRE and (B) denotes different from 510 min infusions PRE. The relative enrichment at 12 month compared to that after 510‐min infusion PRE was 33 ± 2 (SEM)%. Values are mean ± SD.

(A)			
	Background	2 h flood	40 days after flood
Patella tendon protein	1.0888 ± 0.0006	1.0894 ± 0.0008	1.0897 ± 0.0012
Skin protein	1.0888 ± 0.0005		1.0887 ± 0.0013

(B)			
		PRE	12 months later
Myofibrillar protein	90 min infusion	1.0850 ± 0.0007	1.0885 ± 0.0013^ab^
	510 min infusion	1.0961 ± 0.0024^a^	

To emphasize the impact of essential amino acid tracer recycling in the pool of labeled proteins from the myofibrillar protein fraction, we report unpublished data from the study by Moberg and colleagues (Moberg et al. 2016). From Figure 8, it appears that the variation in tracer abundance in the myofibrillar protein pool increases as a function of repeated trials, which we argue is due to tracer‐recycling as a consequence of repeated tracer exposures. In Study 3, we would like to point the attention to the methodological phenomenon as we studied individuals in the recovery from resistance exercise and provided different feeding (water, free amino acids), which are interventions that most likely have impacted the numerical values. However, the resistance exercise was standardized across trials and the feeding regimens were randomized between trials. Further, muscle biopsies were obtained from both legs, alternating within each trial, which we showed in Study 1 add some variability, though evenly across all trials.

In conclusion, the present study demonstrates that the measurement of human myofibrillar protein fractional synthesis – using the direct incorporation, precursor–product approach – and breakdown – using the deuterated water prelabeling approach – rates are determined by similar intrasubject and intersubject coefficient of variation. In comparison, the use of phenylalanine as representative of any essential amino acid tracer for the FBR determination was not successful in the present setting, most likely due to recycling, and hence, more work is required to reveal whether such approach may be feasible. Further, the application of the prelabeling approach to investigate the fractional breakdown rate of a protein pool with a complex post‐translational modification and maturation process, exemplified by the tendinous collagen protein, revealed nonsense enrichments in our setup. This suggests that when multiple pools of protein with very different turnover rates exist, most likely due to the complex maturation processes, variation in prelabeling and/or label‐disappearance kinetics are too divergent and the loss of prelabeling in a matrix homogenate is not homogenous. Although repeated tracer infusions also indicate divergent turnover rates in myofibrillar proteins, the complexity of collagenous protein matrices may be higher, and therefore, more work must be performed to reveal whether the FBR approach can be applied on such tissues before being applied. Therefore, we recommend that when the FBR of protein fractions from other proteins/tissues and even from different species (in vivo or in vitro) is measured, evidence for its applicability should be demonstrated.

## Conflict of Interest

The authors declare no conflict of interests, and there are no financial conflicts to disclose.
